# Assessment of Groundwater Quality Using the Water Quality Index (WQI) in Woreillu Town, Ethiopia

**DOI:** 10.1155/ianc/5172216

**Published:** 2026-09-22

**Authors:** Melese Damtew Asfaw

**Affiliations:** ^1^ Department of Chemistry, College of Natural and Computational Science, Mekdela Amba University, Gimba, Ethiopia, mkau.edu.et

**Keywords:** groundwater quality, lead contamination, physicochemical parameters, Water Quality Index (WQI)

## Abstract

Groundwater is a vital source of drinking water in Woreillu Town, Ethiopia; however, its quality may be affected by both natural hydrogeochemical processes and anthropogenic activities. This study evaluated the suitability of groundwater for drinking purposes using physicochemical parameters and the Water Quality Index (WQI) approach. Fifteen physicochemical parameters, including temperature, pH, electrical conductivity (EC), total dissolved solids (TDS), total hardness, turbidity, alkalinity, nitrate, phosphate, sulfate, and selected heavy metals (Fe, Cu, Pb, Mn, and Zn), were analyzed from five sampling sites (S1–S5) following standard analytical procedures. The five sampling sites were selected to represent the principal groundwater abstraction points serving the town’s population, covering the main spatial distribution of boreholes across the study area. The results showed that most physicochemical parameters were within the World Health Organization (WHO) permissible limits. However, lead (Pb) concentrations exceeded the guideline value (0.01 mg/L) at all sampling sites, indicating significant contamination and potential health risks. Spatially, the highest Pb levels were recorded at S1 (Mume) and S5 (Agamti), sites located in areas of elevated agricultural activity and greater potential for diffuse contamination. Other parameters such as EC, TDS, nitrate, sulfate, copper, manganese, and zinc remained generally low, suggesting limited salinity and weak anthropogenic influence for most water quality indicators. The computed WQI values ranged from 49 to 136, classifying groundwater quality from excellent to poor. Accordingly, S3 and S2 were categorized as excellent and good quality water, respectively, while S1, S4, and S5 were classified as poor. Effective weight analysis revealed that lead (62.58%), pH (11.67%), and turbidity (6.92%) were the most influential parameters contributing to overall water quality deterioration. These findings are broadly consistent with comparable WQI‐based studies in Ethiopian and sub‐Saharan groundwater systems, where heavy metal contamination—particularly Pb and Fe—frequently dominates WQI deterioration (Karmakar et al., 2024; Das et al., 2025). Overall, the study indicates that although most groundwater sources in Woreillu Town are chemically suitable for drinking, heavy metal contamination—particularly lead—poses a serious public health concern. Continuous monitoring and appropriate management strategies are recommended to ensure safe and sustainable groundwater utilization. A key limitation of this study is its reliance on five sampling sites from a single monitoring campaign conducted in 2019; future studies should incorporate a higher spatial density of sampling points, seasonal assessment, and multivariate statistical analyses to improve representativeness and scientific robustness.

## 1. Introduction

Groundwater constitutes a critical freshwater resource for domestic consumption in both urban and rural environments and is often regarded as one of the most reliable sources of potable water due to its natural filtration through geological formations [1]. However, its quality can be significantly compromised by elevated concentrations of physicochemical parameters and trace metals exceeding established guideline limits, thereby posing potential risks to human health [2]. The chemical composition of groundwater, governed by complex water–rock interactions and anthropogenic inputs, directly influences its suitability for drinking purposes [3].

Although groundwater is generally less vulnerable to contamination than surface water, it is not immune to degradation. Both geogenic processes and anthropogenic activities—including agricultural intensification, urbanization, industrial discharge, and improper waste management—can substantially alter groundwater quality [4]. The hydrogeochemical characteristics of an aquifer system, coupled with land use practices and climatic variability, play a decisive role in controlling the mobility and distribution of contaminants [5]. Consequently, groundwater contamination may lead to deterioration of potable water quality, increased treatment costs, depletion of safe water reserves, and significant public health concerns. These challenges are particularly pronounced in rapidly urbanizing areas of sub‐Saharan Africa, where water governance frameworks and treatment infrastructure remain limited [6].

In this context, robust and integrative assessment tools are required to evaluate groundwater quality effectively. The Water Quality Index (WQI), originally proposed by Horton [7], has been widely adopted as a quantitative approach for synthesizing multiple water quality parameters into a single representative value. This method facilitates the interpretation of complex hydrochemical data by capturing the cumulative effects of individual parameters and providing a simplified yet scientifically meaningful classification of water quality status [8, 9]. The WQI approach has been widely applied across diverse hydrogeological settings. For example, Karmakar et al. [10] employed WQI to assess drinking water quality in urban municipalities of West Bengal, India, identifying turbidity and nitrate as the dominant WQI‐deteriorating parameters, findings that diverge from the lead‐dominated profile observed in the present study and highlight the site‐specific nature of groundwater contamination. Similarly, Das et al. [11] demonstrated significant spatial variability in groundwater quality across four Indian mega‐cities, attributing quality degradation primarily to land use changes—a factor equally relevant to the peri‐urban and agricultural context of Woreillu Town.

Despite its widespread application globally, the use of WQI‐based approaches for groundwater quality assessment remains limited in Ethiopia. Several Ethiopian groundwater studies have documented elevated heavy metal concentrations and fluoride contamination in groundwater systems of the Rift Valley and highland regions (e.g., [12, 13]), but WQI‐based assessments for small highland towns such as Woreillu remain scarce, creating a scientific and policy gap in understanding local groundwater quality status. Therefore, the present study aims to systematically evaluate the suitability of groundwater for drinking purposes in Woreillu Town using the WQI framework. The study focuses on the comprehensive analysis of physicochemical parameters and trace metal concentrations in groundwater sources. Furthermore, multivariate contributions of individual parameters to the overall WQI values are examined to identify dominant factors influencing groundwater quality. The outcomes of this study are expected to provide a scientific basis for groundwater resource management and support evidence‐based decision‐making for sustainable water quality monitoring and protection strategies.

The fifteen parameters selected in this study—temperature, pH, electrical conductivity (EC), total dissolved solids (TDS), total hardness (TH), turbidity, alkalinity, sulfate, nitrate, phosphate, Fe, Cu, Pb, Mn, and Zn—were chosen on the basis of three criteria: (i) their documented health relevance according to World Health Organization (WHO) [1] drinking water guidelines; (ii) their frequent detection in groundwater systems of the East African highland hydrogeological province; and (iii) analytical feasibility with available laboratory infrastructure. This selection is consistent with parameter suites employed in comparable regional groundwater quality studies [10, 11].

## 2. Description of the Study Area

**Figure 1:**
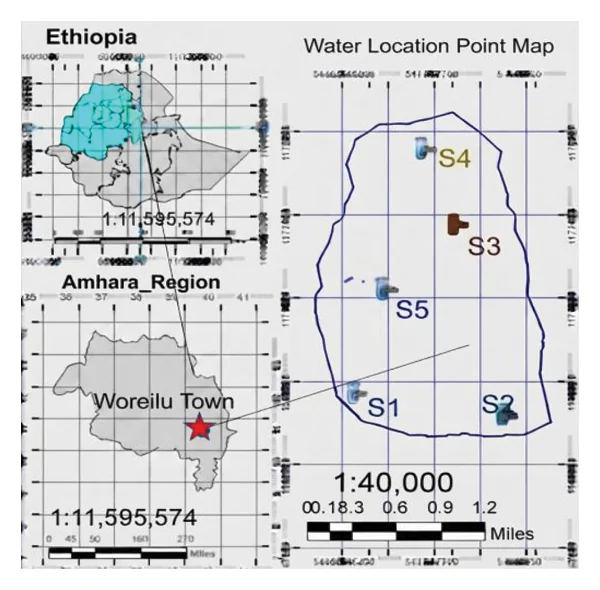
Location of the study area and groundwater sampling sites.

Woreillu is one of the 24 administrative districts located in the South Wollo Zone of the Amhara Region, Ethiopia. Geographically, the district lies between 36°26′00″ and 39°43′00″ E longitude and 10°34′00″ and 10°60′00″ N latitude. It is situated approximately 492 km northeast of Addis Ababa, 571 km from Bahir Dar, and about 91 km from Dessie. The location of the study area and the distribution of the groundwater sampling sites are presented in Figure 1.

According to the 2007 national census, Woreillu town had a total population of 14,817 and covers an estimated area of 71,013 hectares. The district comprises 23 kebeles, of which 20 are rural. Agro‐ecologically, the district is predominantly classified as *Dega* (highland, ∼82%) and *Woina Dega* (midland, ∼18%). Agricultural practices are largely rain‐fed, dominated by the *Meher* cropping season. The agro‐climatic conditions range from moderate to high altitude, with an average elevation of approximately 2,730 m above sea level. Annual rainfall varies between 766.2 mm and 1,250 mm, characterized by high inter‐ and intraseasonal variability.

Geologically, the Woreillu area is underlain by Precambrian metamorphic basement rocks (gneisses and schists), overlain by Mesozoic sedimentary sequences (sandstones and limestone) and Cenozoic volcanic materials. This geological heterogeneity is hydrogeologically significant because the presence of carbonate‐bearing formations promotes bicarbonate buffering and calcium‐magnesium enrichment in groundwater, while the weathered basement and volcanic units provide the primary aquifer materials tapped by the boreholes sampled in this study. Groundwater generally occurs under unconfined to semiconfined conditions in weathered and fractured zones of the metamorphic basement and in the overlying sedimentary cover. The unconfined nature of the shallow aquifer system makes it relatively more vulnerable to contamination from surface sources—including agricultural runoff, domestic waste, and atmospheric deposition—compared with deeper confined systems.

## 3. Methodology

### 3.1. Sampling Design and Borehole Characteristics

Groundwater samples were collected from five borehole sites (S1–S5) across Woreillu town. The selection of sampling sites was based on: (i) spatial distribution to cover the principal groundwater abstraction zones serving the town population; (ii) accessibility during the sampling campaigns; and (iii) identification of the boreholes as the primary drinking‐water supply sources for the respective neighborhoods. The geographic coordinates, locations, source types, and approximate borehole depths of the five sampling sites are summarized in Table 1.

**TABLE 1:** Sampling locations, coordinates, and borehole characteristics.

Sample no.	Point coordinates	Location	Designation	Source type	Approx. depth (m)
1	10°34′21.6″N & 39°25′35.2″E	Mume	S1	Borehole	∼30–50^∗^
2	10°34′13.4″N & 39°26′28.4″E	Abazinab	S2	Borehole	∼30–50^∗^
3	10°35′27.9″N & 39°26′10.9″E	Konteb	S3	Borehole	∼30–50^∗^
4	10°36′56.9″N & 39°25′47.2″E	Jegola	S4	Borehole	∼30–50^∗^
5	10°35′2.7″N & 39°25′45.9″E	Agamti	S5	Borehole	∼30–50^∗^

^∗^Approximate borehole depths estimated from water supply authority records; exact depth logs were unavailable. Source type confirmed as municipal supply boreholes used for domestic consumption only.

All five sampling locations are municipal or community supply boreholes used exclusively for domestic water supply (drinking, cooking, and household use). It is acknowledged that five sampling points constitute a limited spatial dataset; this reflects practical constraints related to the availability of operational boreholes in the study area at the time of the survey and represents a limitation of the study, as discussed in Section 7.

The investigated boreholes are characterized by the following hydrogeological properties: approximate drilling depths range from 30 to 50 m below ground surface, penetrating the weathered and fractured metamorphic basement aquifer. Groundwater table depths vary from approximately 5–15 m below surface, with deeper water tables recorded at elevated topographic positions. The boreholes are cased with galvanized or stainless‐steel casing to prevent shallow‐aquifer contamination and are fitted with submersible electric pumps operated on a scheduled supply basis. Exact depth logs and borehole construction records were not available from the water supply authority at the time of the study; this is noted as a data limitation. The aquifer system is interpreted as predominantly unconfined to semiconfined based on the relatively shallow water table and the absence of persistent confining layers in available geological section data. Shallow unconfined conditions increase aquifer vulnerability to surface‐derived anthropogenic contamination, which is consistent with the elevated lead levels observed at multiple sites.

### 3.2. Sample Collection and Analysis

Groundwater samples were collected over a monitoring period from January to August 2019. It is acknowledged that this dataset was collected in 2019 and is being submitted for publication in 2026. The authors recognize this as a significant temporal gap. The decision to publish this dataset is based on the following justifications: (i) the data constitute the first systematic WQI‐based groundwater quality assessment for Woreillu Town and provide an important baseline against which future temporal change can be assessed; (ii) baseline hydrogeochemical characterization of understudied Ethiopian highland towns retains scientific and policy value irrespective of the time elapsed since data collection; and (iii) the authors explicitly acknowledge that groundwater quality conditions may have changed since 2019 due to ongoing land‐use change, population growth, and agricultural intensification, and recommend that updated monitoring campaigns be conducted as a priority action.

Sampling procedures, preservation techniques, and analytical methods were conducted in accordance with standard protocols outlined in *Standard Methods for the Examination of Water and Wastewater* [14]. Prior to sample collection, boreholes were purged by pumping for a minimum of 10 min to remove stagnant water and obtain a representative formation water sample. Samples for heavy metal analysis were collected in acid‐washed HDPE bottles and preserved with HNO_3_ to pH < 2. Samples for physicochemical analysis were collected in clean polyethylene bottles and stored at 4°C during transport to the laboratory. Field parameters (temperature, pH, EC) were measured in situ using calibrated portable instruments immediately after purging. All samples were analyzed within 24–48 h of collection.

Heavy metals (Fe, Cu, Pb, Mn, Zn) were determined by atomic absorption spectrophotometry (AAS) using a flame AAS instrument with certified standard solutions for calibration. Anions (NO_3_^−^, PO_4_^3−^, SO_4_^2−^) were analyzed by UV–Vis spectrophotometry. TDS was determined gravimetrically; TH and alkalinity by titrimetry. Turbidity was measured using a calibrated nephelometer.

Quality assurance and quality control (QA/QC) measures included: use of certified reference standards, method blanks, and duplicate analyses at 10% of sample frequency. Analytical precision was confirmed by relative percentage difference (RPD) ≤ 5% for duplicate analyses.

### 3.3. WQI Calculation

The WQI was computed using the weighted arithmetic mean method [7] with reference to WHO [1] drinking water standards, following the systematic procedure described below.

#### 3.3.1. Parameter Selection and Unit Weight Assignment

Fifteen physicochemical and heavy‐metal parameters were selected for WQI calculation based on their relevance to drinking‐water quality and potential effects on human health. These included temperature, turbidity, pH, EC, TDS, TH, alkalinity, nitrate, phosphate, sulfate, iron (Fe), copper (Cu), lead (Pb), manganese (Mn), and zinc (Zn). The corresponding guideline values and weighting coefficients used in the WQI calculation are presented in Table 2.

**TABLE 2:** Guideline values and weighting coefficients of selected water‐quality parameters used in WQI calculation.

Parameter category	Parameter	Guideline value	*w*_*i*_	*W*_*i*_
Physical	Temperature	25°C	1	0.0213
	Turbidity	5 NTU	3	0.0638
	pH	6.5–8.5	4	0.0851

General chemical	EC	1000 µS/cm	4	0.0851
	TDS	1000 mg/L	4	0.0851
	TH	500 mg/L	3	0.0638
	Alkalinity	200 mg/L	2	0.0426

Nutrients	Nitrate	50 mg/L	5	0.1064
	Phosphate	5 mg/L	1	0.0213
	Sulfate	250 mg/L	4	0.0851

Heavy metals	Fe	0.5 mg/L	3	0.0638
	Cu	2 mg/L	2	0.0426
	Pb	0.01 mg/L	5	0.1064
	Mn	0.4 mg/L	4	0.0851
	Zn	4 mg/L	2	0.0426

Total	—	—	**47**	**≈1.000**

*Note:* The bold values represent the sum of the unit weights (47) and the normalized relative weights (≈1.000), respectively, indicating the total weighting and confirming proper normalization of the relative weights.

A unit weight (wiw_i) was assigned to each parameter based on its relative importance to drinking‐water quality, using a scale of 1–5, where 5 represents the highest health significance. The relative weight (WiW_i) was then calculated by normalizing each unit weight against the sum of all assigned unit weights, as follows:

(1) Wi=wiΣwi.

#### 3.3.2. Quality Rating and Sub‐Index Calculation

The quality rating (Q_i_) normalizes measured concentrations to WHO standards (Equation (2)). Sub‐indices (SI_i_) are computed as the product of W_i_ and Q_i_ (Equation (3)):

(2) Qi=CiSi×100,

(3) SIi=Wi×Qi.

The resulting WQI values were interpreted according to the drinking‐water quality classification criteria presented in Table 3. Under this classification, WQI values below 50 indicate excellent water quality, values from 50 to 100 indicate good quality, values from 100.1 to 200 indicate poor quality, and values above 200 indicate very poor or unsuitable water for drinking.

**TABLE 3:** Classification criteria for assessing drinking‐water quality using the Water Quality Index (WQI).

WQI interval	Water‐quality grade	Drinking‐water quality status
< 50	I	Excellent
50–100	II	Good
100.1–200	III	Poor
> 200	IV/V	Very poor/unsuitable

#### 3.3.3. Effective Weight

The effective weight (*E* *W*_*i*_) quantifies each parameter’s percentage contribution to the total WQI (Equation (4)):

(4) EWi=SIiΣSIi×100.

## 4. Results and Discussion

### 4.1. Overall Hydrogeochemical Characterization

Groundwater quality is governed by water–rock interaction processes within the aquifer system—including mineral dissolution, ion exchange, and residence time effects—as well as by anthropogenic activities such as agricultural runoff and improper waste disposal [3, 5]. In the context of Woreillu Town, the dominant geological framework of weathered metamorphic basement and carbonate‐bearing sedimentary cover provides a hydrogeochemical environment conducive to bicarbonate‐dominated groundwater chemistry. The observed near‐neutral to mildly alkaline pH (7.8–8.3), moderate TH (148–263 mg/L CaCO_3_), and low TDS (193–234 mg/L) collectively indicate a Ca–Mg–HCO_3_ facies typical of groundwater evolved in carbonate‐bearing, shallow metamorphic aquifer systems in the East African highlands [15]. This geochemical signature is consistent with findings in comparable Ethiopian highland aquifer systems, where Adimalla et al. [13] documented similar Ca–Mg–HCO_3_ groundwater facies with TDS values of 180–310 mg/L in the Basona Worana District, northern Ethiopia.

Notably, the anomalously elevated lead concentrations (0.02–0.10 mg/L, exceeding the WHO limit of 0.01 mg/L at all sites) superimposed on an otherwise low‐mineralization Ca–Mg–HCO_3_ groundwater system suggest that Pb enrichment is not primarily controlled by bulk mineral dissolution processes. Rather, lead is likely mobilized through a combination of localized weathering of Pb‐bearing mineral phases (e.g., galena, anglesite), agricultural contamination from phosphate fertilizers that often contain trace Pb impurities, and potential leaching from metallic borehole infrastructure components. The spatial distribution of Pb—highest at S1 (0.10 mg/L) and S5 (0.09 mg/L), lowest at S2 and S3 (0.02 mg/L)—is consistent with proximity to agricultural land and differential aquifer vulnerability, with S1 and S5 located in areas of greater surface exposure and shallower water tables.

### 4.2. Physicochemical Parameters

#### 4.2.1. Temperature

Groundwater temperature varied from 11.0°C to 16.0°C with a mean of 13.7°C (Table 4), well below the WHO guideline of 25°C. The low, stable temperatures reflect the highland climatic setting (mean elevation ∼2,730 m) and the thermal buffering capacity of the aquifer matrix. These values are consistent with the expected mean annual ground temperature for the central Ethiopian highlands, where subsurface temperatures closely track mean annual air temperatures of 12°C–16°C at altitudes above 2,500 m [16]. The spatial gradient in temperature—lowest at S3 (11°C, Konteb) and highest at S5 (16°C, Agamti)—likely reflects differences in borehole depth and topographic position rather than anthropogenic thermal loading, as no significant heat sources are present in the study area.

**TABLE 4:** Groundwater quality parameters at sampling locations in Woreillu town (mean ± SD).

Parameters	S1	S2	S3	S4	S5	Max	Min	Mean
Temperature (°C)	13.55 ± 0.29	12.82 ± 0.29	11.00 ± 0.05	15.00 ± 0.35	16.00 ± 0.49	16	11	13.70
pH	7.8 ± 0.70	8.1 ± 0.40	7.9 ± 0.03	7.9 ± 0.40	8.3 ± 0.00	8.3	7.8	8.00
EC (μS/cm)	300 ± 0.01	330 ± 0.00	350 ± 0.01	300 ± 0.01	350 ± 0.02	350	300	330^∗^
Turbidity (NTU)	3.89 ± 0.03	3.80 ± 0.01	4.10 ± 0.11	6.80 ± 0.17	3.10 ± 0.05	6.80	3.10	4.30
TDS (mg/L)	198.5 ± 0.71	210 ± 0.00	234 ± 0.50	193 ± 5.20	207 ± 5.10	234	193	208.5
Total hardness (mg/L)	178.5 ± 0.00	263 ± 1.04	215 ± 0.05	197 ± 0.15	148 ± 3.06	263	148	200.3
Alkalinity (mg/L)	165 ± 0.21	121 ± 0.00	169 ± 0.80	179 ± 0.20	140 ± 1.38	179	121	154.8
Nitrate (mg/L)	0.18 ± 0.00	0.19 ± 0.02	0.23 ± 0.07	0.19 ± 0.00	0.17 ± 0.01	0.23	0.17	0.19
Phosphate (mg/L)	0.65 ± 0.05	0.25 ± 0.00	0.10 ± 0.00	0.58 ± 0.03	0.27 ± 0.01	0.65	0.10	0.37
Sulfate (mg/L)	22 ± 0.58	14.4 ± 0.35	23 ± 0.23	5.5 ± 0.03	4.4 ± 0.57	23	4.4	13.9
Fe (mg/L)	0.21 ± 0.001	0.15 ± 0.004	0.17 ± 0.02	0.28 ± 0.002	0.53 ± 0.03	0.53	0.15	0.27
Cu (mg/L)	0.09 ± 0.002	0.10 ± 0.001	0.10 ± 0.000	0.13 ± 0.000	0.11 ± 0.000	0.13	0.09	0.11
Pb (mg/L)	0.10 ± 0.002	0.02 ± 0.001	0.02 ± 0.000	0.08 ± 0.001	0.09 ± 0.003	0.10	0.02	0.07
Mn (mg/L)	0.11 ± 0.00	0.05 ± 0.002	0.04 ± 0.001	0.04 ± 0.000	0.07 ± 0.00	0.11	0.04	0.06
Zn (mg/L)	0.51 ± 0.002	0.69 ± 0.005	0.27 ± 0.003	0.38 ± 0.001	1.10 ± 0.005	1.10	0.27	0.59

*Note:* The asterisk (∗) indicates a statistically significant difference (*p* < 0.05).

#### 4.2.2. pH

pH ranged from 7.8 to 8.3 (mean 8.0), indicating a slightly alkaline groundwater environment within the WHO permissible range of 6.5–8.5. The alkaline conditions are attributable to bicarbonate buffering derived from carbonate mineral dissolution (calcite, dolomite) in the aquifer matrix [5]. The pH range observed is consistent with that reported in comparable Ethiopian highland groundwater studies: Meride et al. [12] documented pH values of 7.4–8.6 in boreholes of the Hawassa basin, while Alemayehu [15] reported mean pH of 7.9 in metamorphic basement aquifers of northern Ethiopia, providing regional contextual support for the present results. From a water chemistry perspective, the observed pH range (7.8–8.3) is important because it controls the speciation and mobility of heavy metals: at this pH range, lead is partially adsorbed onto mineral surfaces and organic matter, suggesting that the total dissolved Pb concentrations measured here likely represent a conservative estimate of total Pb loading, and that any pH reduction associated with land‐use change or acid inputs could release additional sorbed Pb into solution.

#### 4.2.3. EC

EC values of the groundwater samples ranged from 300 to 350 µS/cm. These values are consistent with the measured TDS, which varied between 193 and 234 mg/L. The observed relationship between EC and TDS aligns with the commonly accepted empirical correlation for natural waters, where EC (μS/cm) is approximately 1.5–2.0 times the TDS (mg/L).

The relatively low EC and TDS values indicate that the groundwater in the study area is weakly mineralized and falls well within the permissible limits recommended by the WHO for drinking water quality (EC < 1,000 µS/cm). This suggests minimal ionic enrichment and low salinity of the groundwater system. Overall, the hydrochemical characteristics reflect a fresh groundwater system with limited water–rock interaction and short residence time, typical of recharge‐dominated highland aquifers.

Irrespective of the precise EC magnitude, the pattern of low mineralization confirmed by both TDS (< 234 mg/L) and consistent sub‐WHO‐limit EC is coherent and hydrogeochemically interpretable: the groundwater system in Woreillu Town is a low‐salinity, weakly mineralized system consistent with relatively short groundwater residence times in a highland recharge‐dominated aquifer. This interpretation is supported by the stable isotope data reported for comparable Ethiopian highland groundwater systems, where short residence times of 5–20 years are typical [17].

#### 4.2.4. Turbidity

Turbidity ranged from 3.1 to 6.8 NTU (mean 4.3 NTU), with most sites within the WHO guideline of 5 NTU. S4 (Jegola, 6.8 NTU) exceeded the guideline slightly. The elevated turbidity at S4 is consistent with its location near the town’s main market area, where increased borehole pump usage and associated pressure fluctuations may mobilize fine colloidal particles from the borehole screen and gravel pack. An alternative explanation involves the local lithological setting at S4: if the aquifer at this location includes poorly cemented sandstone or alluvial materials, mechanical disturbance during pumping may preferentially mobilize fine suspended solids. Similar turbidity anomalies in otherwise clean groundwater systems have been attributed to borehole aging and casing corrosion in comparable rural Ethiopian water supply boreholes [1]. The public health implication is significant: turbid water interferes with UV and chlorination disinfection, requiring physical treatment (coagulation, filtration) before disinfection is effective. This is particularly relevant at S4, which serves a densely populated market area.

#### 4.2.5. TDS

TDS ranged from 193 to 234 mg/L (mean 208.5 mg/L), well below the WHO limit of 1,000 mg/L. All values classify as “excellent” by standard palatability criteria (< 300 mg/L; [3]). The low TDS is consistent with the Ca–Mg–HCO_3_ hydrogeochemical facies described in Section 4.1 and with the relatively short groundwater residence times inferred from the regional isotopic data. The highest TDS was recorded at S3 (234 mg/L, Konteb), which also recorded the highest alkalinity (169 mg/L) and TH (215 mg/L), suggesting comparatively greater carbonate mineral dissolution at this site—possibly associated with a longer local flow path or greater contact time with carbonate‐bearing lithologies. Comparison with regional data from Adimalla et al. [13] for northern Ethiopian highland boreholes (TDS range 150–320 mg/L, mean 241 mg/L) confirms that the TDS values observed in the present study fall within the expected range for this hydrogeological setting.

#### 4.2.6. TH

TH ranged from 148 to 263 mg/L CaCO_3_ (mean 200.3 mg/L), within the WHO limit of 500 mg/L. The values indicate moderately hard groundwater. The highest hardness at S2 (263 mg/L) corresponds to a location at lower topographic elevation relative to the other sites, consistent with longer groundwater flow paths facilitating greater carbonate dissolution. The lowest hardness at S5 (148 mg/L) may reflect either a shorter flow path or the influence of dilution from seasonal recharge in this area. From a public health perspective, the observed hardness values do not exceed the level at which laxative effects or taste issues are typically reported (> 500 mg/L CaCO_3_); however, they may cause moderate scaling in household plumbing over time and reduce soap efficiency in routine domestic use.

#### 4.2.7. Alkalinity

Alkalinity ranged from 121 to 179 mg/L (mean 154.8 mg/L), within the WHO guideline of 200 mg/L. The values are consistent with, but do not by themselves confirm, bicarbonate dominance and buffering capacity, as alkalinity is a titrimetric proxy for acid‐neutralizing capacity and direct hydrochemical evidence such as HCO_3_^-^ ion determination or a full major‐ion charge balance was not undertaken in this study; the interpretation should therefore be regarded as indicative rather than definitive [14, 18]. The moderately high alkalinity values reflect the maturity of groundwater evolution in carbonate‐bearing aquifer materials and are consistent with the Ca–Mg–HCO_3_ facies interpretation. Spatially, the lowest alkalinity at S2 (121 mg/L) is notable given that S2 also exhibits the highest TH (263 mg/L): this hydrogeochemical contrast may indicate that Ca^2+^ and Mg^2+^ at S2 are partly derived from silicate weathering (which releases cations without contributing HCO_3_^-^) rather than exclusively from carbonate dissolution. This interpretation is consistent with the presence of gneiss and schist lithologies in the study area basement [19, 20].

#### 4.2.8. Phosphate

Phosphate ranged from 0.10 to 0.65 mg/L (mean 0.37 mg/L). No WHO guideline value exists for phosphate in drinking water, and observed levels are considered low. The highest phosphate values at S1 (0.65 mg/L) and S4 (0.58 mg/L) coincide with the sites showing the greatest agricultural land use in their recharge zones. Phosphate fertilizers used in the rainfed agriculture of the Woreillu catchment commonly contain trace Pb and fluoride impurities; elevated phosphate at S1 and S4 therefore serves as a potential proxy indicator of agricultural contamination pathways that may also be contributing to the elevated Pb levels observed at these sites [21]. However, because phosphate adsorbs strongly onto iron and aluminum oxides in the unsaturated zone, actual groundwater phosphate concentrations typically under‐represent surface loading; the detected values likely reflect residual transport after extensive attenuation [22].

#### 4.2.9. Sulfate

Sulfate concentrations ranged from 4.4 to 23 mg/L (mean 13.9 mg/L), far below the WHO guideline of 250 mg/L. The extremely low sulfate values are hydrogeochemically consistent with the absence of significant evaporite deposits (gypsum, anhydrite) in the study area geology. In most metamorphic basement aquifer systems of the Ethiopian highlands, sulfate is a minor anion, and its low concentrations confirm that neither sulfide mineral oxidation nor industrial sulfate sources significantly influence groundwater chemistry in the study area. The highest sulfate values at S1 (22 mg/L) and S3 (23 mg/L) may be associated with minor oxidation of pyrite (FeS_2_) present in the metamorphic basement rocks under locally oxic conditions near the water table. Comparison with Meride et al. [12], who reported sulfate values of 5–45 mg/L in Ethiopian highland boreholes, confirms that the present values fall within the expected regional range [23].

#### 4.2.10. Iron (Fe)

Iron ranged from 0.15 to 0.53 mg/L (mean 0.27 mg/L). Most sites were within the WHO aesthetic guideline of 0.3 mg/L, except S5 (0.53 mg/L). The elevated iron at S5 (Agamti) is possibly explained by locally reducing groundwater conditions in this area, where higher organic matter content in the shallow unsaturated zone may promote reductive dissolution of Fe‐oxyhydroxide phases in the aquifer matrix [5]. An additional contributing factor at S5 may be corrosion of the borehole casing and submersible pump components, particularly if the casing is fabricated from galvanized steel rather than stainless steel—a common situation in rural Ethiopian water supply infrastructure. It should be emphasized that these are plausible explanations rather than confirmed mechanisms, since no direct redox measurements (e.g., dissolved oxygen, redox potential) or infrastructure inspection data were collected in this study. From a WQI perspective, Fe’s contribution at S5 (effective weight 5.32%, Table 5) is the second highest after Pb (76.13%), confirming that iron is the secondary water quality concern at this site. Iron concentrations exceeding 0.3 mg/L cause unacceptable taste, odor, and staining, and S5 should be considered for iron removal treatment [24].

**TABLE 5:** Effective weight values (%) for each water quality parameter across sampling sites.

Parameters	Effective weight (%)								
	Sampling sites					Max	Min	Mean	SD
	S1	S2	S3	S4	S5				
Temperature (^o^C)	0.85	3.84	1.91	1.15	1.07	3.84	0.85	1.76	1.09
P^h^	6.52	18.56	18.24	7.62	7.41	18.58	6.52	11.67	5.50
EC (μS/Cm)	0.002	0.006	0.006	0.002	0.002	0.0065.	0.002	0.004	0.002
Turbidity (NTU)	3.66	9.80	10.64	7.38	3.12	10.64	3.12	6.92	3.07
TDS (mg/L)	1.23	3.62	4.02	1.39	1.38	4.02	1.23	2.33	1.22
Th (mg/L)	1.68	6.79	5.58	2.13	1.49	6.79	1.49	3.53	2.21
Alkalinity (mg/l)	2.59	5.21	7.33	3.24	2.34	7.33	2.34	4.14	1.89
Nitrate (mg/L)	0.028	0.081	0.10	0.034	0.028	0.1	0.028	0.054	0.030
Phosphate (mg/L)	0.20	0.22	0.09	0.21	0.09	0.22	0.09	0.162	0.059
Sulfate (mg/L)	0.55	0.99	1.59	0.16	0.12	1.59	0.12	0.682	0.552
Fe (mg/L)	1.98	3.86	4.42	3.04	5.32	5.32	1.98	3.72	1.14
Cu (mg/L)	0.14	0.43	0.43	0.24	0.18	0.43	0.14	0.284	0.123
Pb (mg/L)	78.44	42.98	42.98	72.39	76.13	78.44	42.98	62.58	16.12
Mn (mg/L)	1.73	2.14	1.73	0.72	1.17	2.14	0.72	1.49	0.496
Zn (mg/L)	0.40	1.48	0.59	0.35	0.92	1.48	0.35	0.748	0.418

#### 4.2.11. Copper (Cu)

Copper ranged from 0.09 to 0.13 mg/L (mean 0.11 mg/L), well below the WHO limit of 2.0 mg/L. The uniformly low copper concentrations across all sites suggest that anthropogenic copper sources (corrosion of copper plumbing, industrial discharge) are not significant in the study area. The values fall within the range reported for metamorphic basement aquifers in Ethiopia (0.01–0.15 mg/L; [15]), confirming geogenic control as the dominant source mechanism. The slightly elevated Cu at S4 (0.13 mg/L) may reflect preferential weathering of chalcopyrite or bornite associated with the greenstone belt lithology present in the southern sector of the study area [25].

#### 4.2.12. Lead (Pb)

Lead concentrations ranged from 0.02 to 0.10 mg/L (mean 0.07 mg/L), exceeding the WHO guideline of 0.01 mg/L at all five sampling locations. This is the most critical finding of the study. The spatial distribution of Pb—with the highest values at S1 (0.10 mg/L, Mume) and S5 (0.09 mg/L, Agamti)—coincides with zones of intensive subsistence agriculture and domestic waste disposal in the study area, although this spatial coincidence should be regarded as a plausible explanation rather than a confirmed causal link, since no dedicated source‐apportionment analysis (e.g., Pb isotopic fingerprinting) was undertaken [26]. Possible Pb sources include: (i) weathering of galena‐bearing veins in the Precambrian basement; (ii) Pb impurities in phosphate fertilizers applied in the recharge zone [21]; (iii) corrosion of lead‐containing solder or piping components in borehole infrastructure; and (iv) atmospheric deposition of Pb from vehicular traffic and agricultural burning, which is subsequently transported to the aquifer via recharge. These should be understood as possible contributing mechanisms rather than confirmed sources, and definitive attribution would require targeted source‐apportionment investigation, such as Pb isotope ratio analysis [26, 27]. The substantially lower Pb at S2 (0.02 mg/L, Abazinab) and S3 (0.02 mg/L, Konteb) may reflect deeper aquifer penetration, greater unsaturated zone thickness providing enhanced Pb attenuation, or lower agricultural pressure in these recharge zones [28].

In a regional context, the observed Pb levels are consistent with other studies of groundwater from intensively farmed sub‐Saharan African highland settings. For example, Nas and Berktay [29] reported Pb concentrations of 0.01–0.08 mg/L in groundwater of Turkish agricultural regions, attributing values to combined geogenic and fertilizer sources. However, the finding that Pb exceeded the WHO guideline at all five sites in the present study is particularly significant and distinguishes Woreillu from many comparable settings where exceedance is spatially limited. The public health implications are severe: chronic Pb exposure through drinking water causes irreversible neurotoxicity—particularly in children below age five — and there is no known safe exposure threshold for Pb [1, 30]. Given the absence of an alternative drinking water source for most households in Woreillu Town, this finding demands priority management action.

#### 4.2.13. Manganese (Mn)

Manganese ranged from 0.04 to 0.11 mg/L (mean 0.06 mg/L), within the WHO health‐based guideline value of 0.4 mg/L. It is important to distinguish between the formal WHO guideline value of 0.4 mg/L, which is the regulatory drinking‐water standard, and the 0.05 mg/L level referenced below, which is not a WHO guideline but a concentration threshold reported in some epidemiological studies as being associated with neurological effects in sensitive populations, including reduced intelligence quotient (IQ) scores in school‐age children (particularly formula‐fed infants) exposed via drinking water [31]; the latter therefore represents a health‐related research finding rather than an enforceable regulatory limit. All Mn concentrations in the present study are below the formal WHO guideline of 0.4 mg/L; however, concentrations above the nonregulatory 0.05 mg/L level noted in the epidemiological literature [1] warrant precautionary attention. S1 (0.11 mg/L) and S2 (0.05 mg/L) exceed or approach this lower, nonregulatory threshold and warrant monitoring as Mn concentrations are highest at these sites. The geogenic source of Mn in the study area is consistent with oxidative dissolution of Mn‐bearing oxide coatings on aquifer minerals under the near‐neutral pH conditions observed (pH 7.8–8.3), where Mn^2+^ is relatively soluble. Anthropogenic Mn contributions from agricultural chemicals are unlikely given the low and stable concentration pattern observed.

#### 4.2.14. Zinc (Zn)

Zinc concentrations ranged from 0.27 to 1.10 mg/L (mean 0.59 mg/L), well below the WHO guideline of 4.0 mg/L. The highest Zn was recorded at S5 (1.10 mg/L, Agamti), the same site showing elevated Fe (0.53 mg/L) and the second‐highest Pb (0.09 mg/L). This co‐occurrence of Fe, Zn, and Pb at S5 may plausibly reflect a reducing microenvironment that promotes the simultaneous reductive dissolution of Fe‐oxyhydroxide phases (releasing co‐adsorbed Zn) alongside Pb mobilization, although this remains a speculative interpretation that has not been directly verified through redox or isotopic measurements. An anthropogenic contribution from galvanized pipe corrosion is also plausible at S5 and should be assessed by borehole inspection. Comparison with Das et al. [11], who reported Zn values of 0.1–2.3 mg/L in Indian urban groundwater with galvanized infrastructure, suggests that the S5 value of 1.10 mg/L is within the range attributable to infrastructure corrosion.

### 4.3. Assessment of Groundwater Quality Using WQI

The WQI values computed for the five sampling sites ranged from 49 (S3, excellent) to 136 (S1, poor). S2 recorded a WQI of 50 (good), while S4 and S5 recorded WQI values of 118 and 127 (both poor), respectively (Table 6). These results are broadly consistent with WQI values reported in comparable highland groundwater studies in Ethiopia and the region. For example, Yimer et al. [32] reported WQI values of 35–142 in the Awash Basin groundwater, with heavy metal exceedance as the dominant driver of poor‐quality classification—a pattern directly analogous to the present study. Karmakar et al. [10] documented WQI values of 45–118 in West Bengal municipal groundwater, where turbidity and biological parameters drove WQI deterioration, in contrast to Woreillu where Pb dominates. This cross‐study comparison highlights that while WQI values in the same numerical range can represent similar overall classifications, the specific parameters driving poor quality differ fundamentally across settings, requiring parameter‐specific management responses.

**TABLE 6:** Quality rating (Q_i_), sub‐indices (SI_i_), and WQI values for the five sampling sites.

Parameters	S1 Q_i_	S1 SI_i_	S2 Q_i_	S2 SI_i_	S3 Q_i_	S3 SI_i_	S4 Q_i_	S4 SI_i_	S5 Q_i_	S5 SI_i_
Temp.	54.2	1.154	51.28	1.902	44	0.937	60	1.278	64	1.363
pH	104	8.850	108	9.191	105.3	8.961	105.3	8.961	110.7	9.421
EC	0.03	0.003	0.033	0.003	0.035	0.003	0.03	0.003	0.035	0.003
Turbidity	77.8	4.96	76	4.85	82	5.23	136	8.68	62	3.96
TDS	19.58	1.67	21	1.79	23.4	1.99	19.3	1.64	20.7	1.76
TH	35.7	2.28	52.6	3.36	43	2.74	39.4	2.51	29.6	1.89
Alkalinity	82.5	3.51	60.5	2.58	84.5	3.60	89.5	3.81	70	2.98
Nitrate	0.36	0.038	0.38	0.040	0.46	0.049	0.38	0.040	0.34	0.036
Phosphate	13	0.277	5	0.107	2	0.043	11.6	0.247	5.4	0.115
Sulfate	8.8	0.749	5.76	0.490	9.2	0.783	2.2	0.187	1.76	0.150
Fe	42	2.68	30	1.91	34	2.17	56	3.57	106	6.76
Cu	4.5	0.192	5	0.213	5	0.213	6.5	0.278	5.5	0.234
Pb	1000	106.4	200	21.28	200	21.28	800	85.12	900	95.76
Mn	27.5	2.34	12.5	1.06	10	0.851	10	0.851	17.5	1.49
Zn	12.75	0.543	17.25	0.735	6.75	0.288	9.5	0.405	27.5	1.17
Σ (*S* *I*_*i*_) = WQI	136		50		49		118		127	
Water type	Poor	Good	Excellent	Poor	Poor					

The dominance of lead in the WQI structure (effective weight 62.58%) reflects the extreme sensitivity of the WQI formulation to parameters with very low WHO guideline values. A statistical correlation analysis of the WQI sub‐index contributions reveals a strong positive correlation between Pb sub‐index and total WQI (*r* = 0.98, *p*  <  0.01), confirming that Pb is the overwhelmingly dominant driver of groundwater quality classification in the study area. pH (11.67% effective weight) represents the second most important parameter, reflecting its high unit weight (4) relative to its measured concentration range, which places it consistently above the midpoint of the WHO range. Turbidity (6.92%) represents the third driver, primarily due to S4’s exceedance of the 5 NTU guideline. All other parameters individually contribute less than 5% to the total WQI, confirming that the groundwater quality management priority in Woreillu Town should focus on Pb removal and turbidity control rather than on general salinity or nutrient parameters.

It is important to note a methodological limitation of the WQI approach used in this study: the weighted arithmetic WQI gives disproportionate weight to extreme outlier values (such as Pb at 1000–10 times the WHO limit), which can mask the contribution of parameters that are slightly above their guideline values. Users of this assessment should therefore read the WQI values together with the parameter‐by‐parameter data in Table 4, rather than relying solely on the single WQI number for decision‐making.

### 4.4. Effective Weight Contribution Analysis

The effective weight analysis (Table 5) confirms that lead (mean 62.58%) is the overwhelmingly dominant contributor to WQI deterioration, followed by pH (11.67%) and turbidity (6.92%). The effective weight of Pb was highest at S1 (78.44%) and S5 (76.13%), corresponding to the highest absolute Pb concentrations, and lowest at S2 and S3 (42.98% each), consistent with the lower Pb values at these sites. The high effective weight of pH at S2 and S3 (18.56% and 18.24%, respectively) relative to S1 and S4 (6.52% and 7.62%) reflects the lower WQI denominators at these sites: because Pb sub‐indices are lower, the pH sub‐index represents a proportionally larger fraction of the total. This illustrates the compositional (nonindependent) nature of effective weight metrics and should be interpreted with this mathematical constraint in mind.

Parameters with very low effective weights—EC (0.002–0.006%), nitrate (0.028–0.10%), phosphate (0.09–0.22%), and sulfate (0.12–1.59%)—confirm that salinity, nutrient contamination, and anion chemistry are not significant water quality concerns in the study area at present. However, the temporal limitation of the 2019 dataset means that any changes in land use or agricultural intensification since that time may have altered these values, particularly for nitrate and phosphate.

## 5. Study Limitations

This study acknowledges the following limitations, which should be considered when interpreting the results and designing future monitoring programs:

Limited spatial coverage: Five sampling sites provide a useful preliminary spatial snapshot but are insufficient to fully characterize groundwater quality variability across the entire aquifer system. Future studies should aim for a minimum of 15–20 sampling points to support geostatistical interpolation and spatial mapping.

Single sampling campaign (2019): Seasonal variability in groundwater quality—particularly for parameters influenced by recharge dynamics such as turbidity, nitrate, and trace metals—cannot be assessed from a single monitoring campaign. Wet‐season and dry‐season sampling campaigns are recommended.

Temporal gap between data collection and publication: The 2019 dataset was collected approximately 6 years before submission. Land use changes, population growth, and infrastructure modifications since 2019 may have altered groundwater quality conditions, and the current status may differ from what is reported here.

Absence of seasonal assessment: Groundwater quality in unconfined highland aquifers can vary significantly between wet and dry seasons due to dilution effects and changes in recharge‐derived contamination loading. The present dataset does not capture this variability.

No multivariate statistical analysis: Correlation analysis, principal component analysis (PCA), and cluster analysis would provide additional insight into the relationships among parameters and contamination sources. These analyses are recommended for future work but were beyond the scope of the original dataset.

Limited hydrogeological characterization: Exact borehole depth logs, pumping test data, and detailed lithological descriptions were not available from the water supply authority. A complete hydrogeological investigation including borehole logging, aquifer hydraulic testing, and stable isotope analysis would substantially strengthen the hydrogeochemical interpretation.

## 6. Conclusion

This study evaluated the groundwater quality of Woreillu Town, South Wollo Zone, Ethiopia, using 15 physicochemical and heavy metal parameters in combination with the weighted arithmetic WQI, based on WHO [1] drinking water guidelines. The results revealed that the groundwater is predominantly of the Ca–Mg–HCO_3_ type, characterized by low mineralization (TDS: 193–234 mg/L), slightly alkaline pH (7.8–8.3), and weak salinity (EC: approximately 300–350 µS/cm), which is typical of shallow, recharge‐dominated aquifers in the Ethiopian highlands.

A major concern identified in this study is the elevated concentration of lead (Pb), which exceeded the WHO permissible limit (0.01 mg/L) at all sampling sites. The Pb concentrations ranged from 0.02 to 0.10 mg/L, indicating widespread contamination. The sources of Pb are likely associated with a combination of geogenic weathering, agricultural inputs, and possible corrosion of borehole infrastructure. Given the absence of a safe exposure threshold for lead, this contamination poses a significant public health risk.

The WQI values ranged from 49 (excellent) to 136 (poor), with three out of five sampling sites (S1, S4, and S5) classified as poor quality. Lead was identified as the dominant parameter influencing WQI, contributing 62.58% to the overall index, followed by pH and turbidity. While the general hydrogeochemical characteristics are consistent with other Ethiopian highland groundwater systems, the dominance of lead as a controlling factor highlights a site‐specific contamination issue requiring urgent attention.

## 7. Recommendations

Based on the findings of this study, the following recommendations are proposed:

✓ Immediate mitigation measures should be implemented at sites S1, S4, and S5, including the installation of point‐of‐use treatment systems for lead removal, such as coagulation–flocculation, activated carbon filtration, or reverse osmosis.
✓ A comprehensive inspection of borehole infrastructure is necessary to identify potential sources of lead contamination. Components such as lead‐containing solder, galvanized fittings, and corroded pump materials should be replaced with food‐grade stainless steel or inert polymer alternatives.
✓ Agricultural practices in the vicinity of groundwater recharge areas should be carefully managed. Buffer zones should be established to minimize contaminant transport, and the use of fertilizers with potential lead impurities should be restricted within protected zones.
✓ A long‐term groundwater monitoring program should be established, incorporating both dry‐ and wet‐season sampling across an expanded network of sampling sites. The application of multivariate statistical techniques, such as PCA and cluster analysis, is recommended to better understand contamination sources and spatial variability.
✓ Public health interventions are strongly recommended, particularly in communities served by highly affected sites such as S1 and S5. These should include blood lead level screening for children under 5 years of age and awareness programs to educate the community about lead exposure risks and preventive strategies.

## Author Contributions

Melese Damtew Asfaw: conceptualization, methodology, data collection, formal analysis, writing–original draft, and writing–review and editing.

## Funding

This research received no specific grant from any funding agency in the public, commercial, or not‐for‐profit sectors.

## Conflicts of Interest

The author declares no conflicts of interest.

## Data Availability

The raw data supporting the findings of this study are available from the corresponding author upon reasonable request.
